# Comparison between the antimicrobial activity of colistin and ceftazidime-avibactam against multidrug-resistant *Pseudomonas aeruginosa* isolated from patients with ventilator-associated pneumonia in intensive care units of Kasr Al-Ainy hospitals

**DOI:** 10.1186/s12879-026-12991-7

**Published:** 2026-03-24

**Authors:** Nahla Yassin Sahloul, Somaia Sayed Helmy, Alaa Reda Awad, Ahmed Ibrahim Elsayed, Mai A. Sahbal, Alaa Mohamed Ibrahim Aboutaleb

**Affiliations:** 1 Department of Microbiology, Faculty of Medicine, Cairo University, Cairo, 11562 Egypt; 2 Department of Critical Care Unit, Faculty of Medicine, Cairo University, Cairo, 11562 Egypt

**Keywords:** VAP, MDR *P. aeruginosa*, Colistin, CAZ/AVI

## Abstract

**Background:**

With the growing challenge of antibiotic resistance, evaluating the effectiveness of existing antimicrobials is crucial. This work aimed to compare the antimicrobial activity of colistin versus ceftazidime/avibactam (CAZ/AVI) against MDR *P. aeruginosa*. It also aimed to estimate the prevalence of *P. aeruginosa* as a causative agent for VAP and the rate of its multi-drug resistance.

**Methods:**

This observational cross-sectional study was conducted from January to June 2025 on ICU patients with VAP at Kasr Alainy Hospitals. A total of 120 endotracheal aspirates were collected from patients and cultured. *P. aeruginosa* was identified, tested for antibiotic susceptibility by the Kirby-Bauer disc diffusion method, and underwent colistin minimal inhibitory concentration (MIC) testing via broth microdilution and CAZ/AVI MIC determination using E-test, following the Clinical and Laboratory Standards Institute 2025 guidelines.

**Results:**

Of 120 ICU patients diagnosed with VAP, *P. aeruginosa* was isolated from 47 (39.2%). Among these, 40 isolates (85.1%) were identified as MDR. Of 40 MDR *P. aeruginosa* isolates, 32 (80%) were susceptible to colistin, with MIC₅₀ and MIC₉₀ values of 2 µg/mL and 4 µg/mL, respectively. In contrast, only 5 (12.5%) isolates were susceptible to CAZ/AVI, with both MIC₅₀ and MIC₉₀ exceeding 16 µg/ml, indicating widespread resistance. The difference in susceptibility between the two antibiotics was statistically significant (*p* **<** 0.01).

**Conclusions:**

Colistin showed significantly greater in vitro effectiveness than CAZ/AVI against MDR *P. aeruginosa* from VAP patients in the ICU, supporting its continued use despite nephrotoxicity concerns. The limited activity of CAZ/AVI underscores the need for ongoing local antimicrobial surveillance and strict stewardship practices.

**Supplementary Information:**

The online version contains supplementary material available at 10.1186/s12879-026-12991-7.

## Introduction

**Fig. 1:**
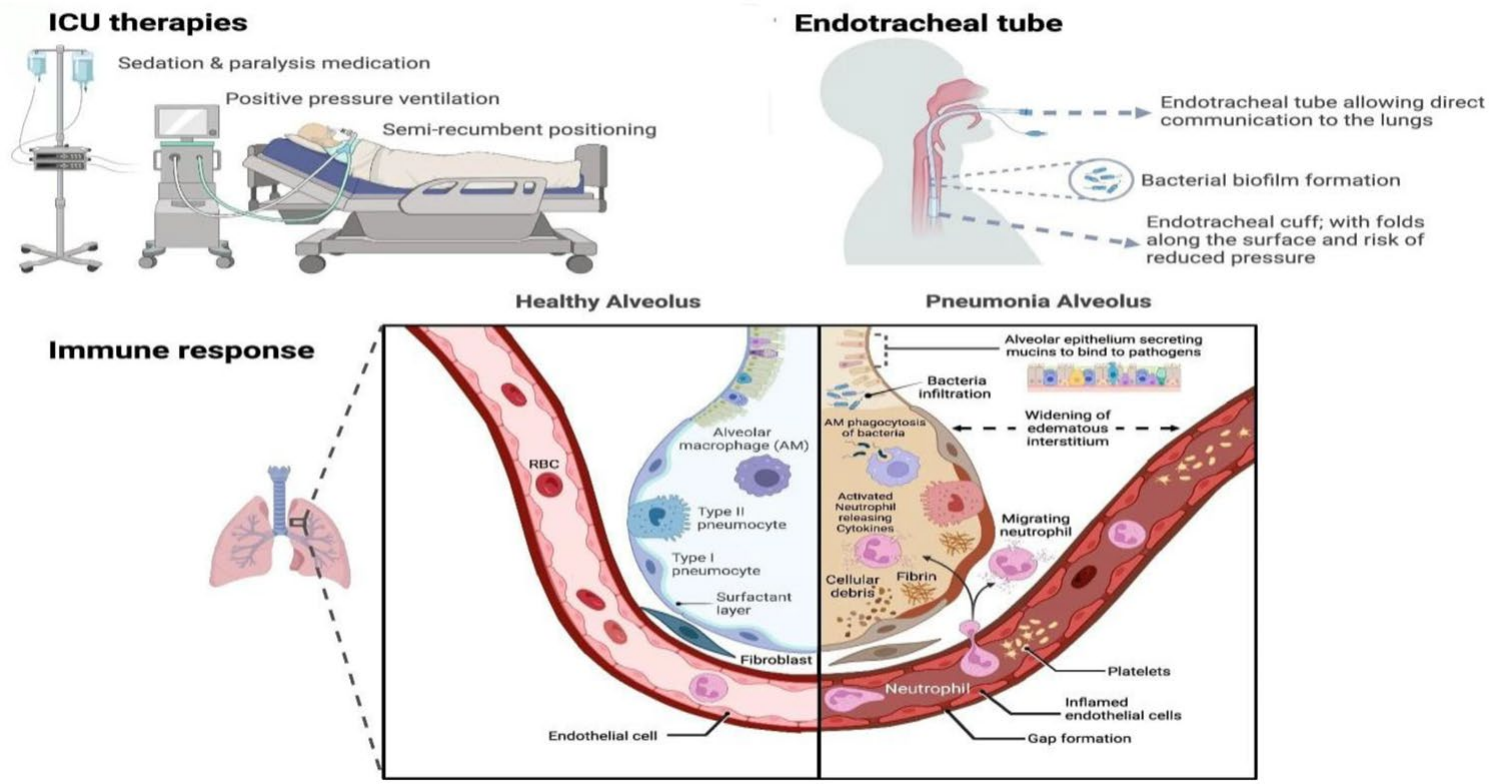
Pathophysiology of VAP [4]

 Ventilator-associated pneumonia (VAP) remains one of the most serious healthcare-associated infections (HAIs) encountered in ICUs, posing a significant threat to critically ill patients. It is defined by the CDC 2025 as pneumonia where the patient is on mechanical ventilation for > 2 consecutive calendar days on the date of event, with day of ventilator placement being Day 1, and the ventilator was in place on the date of event or the day before [1]. VAP is a major ICU infection with higher incidence in low- and middle-income countries (up to ~ 24/1,000 ventilator-days) and lower rates in high-income settings, with mortality ranging from 24 to 76% [2]. Key risk factors include intubation with aspiration of secretions, older age, smoking, comorbidities, impaired consciousness, prolonged ventilation, prior antibiotics, and invasive devices [3]. Early-onset VAP typically involves antibiotic-sensitive organisms (*Streptococcus pneumoniae*,* Haemophilus influenzae*, Methicillin-sensitive *Staphylococcus aureus (MSSA)*(13.6%), and susceptible *Enterobacterales* (14.1%)). In contrast, late-onset VAP is dominated by multidrug-resistant pathogens, including *Pseudomonas aeruginosa* (22.4%), *Acinetobacter baumannii* (35.5%), *Methicillin-resistant Staphylococcus aureus (MRSA)* (44.4%), and resistant *Enterobacterales*; fungi such as *Aspergillus* may occur in debilitated patients [4, 5]. Pathophysiology of VAP centers on the endotracheal tube disrupting airway defenses through microaspiration, biofilm formation, impaired mucociliary clearance, and ventilator-induced inflammation, which together promote colonization and lung injury [6] Fig. 1.

According to modified criteria from CDC 2024, VAP diagnosis requires combining clinical signs (fever, leukocytosis/leukopenia, increased secretions or respiratory decline), new or progressive infiltrates on imaging, and supportive positive quantitative or semi-quantitative culture from bronchoalveolar lavage (BAL) or endotracheal aspirates, as no single gold standard exists [6]. Management begins with early empiric antibiotics covering *S. aureus*, *P. aeruginosa*, and Gram-negative bacilli, adding vancomycin or linezolid if *MRSA* risk is high, and adjusting therapy after 48–72 h; newer options like ceftolozane/tazobactam, ceftazidime/avibactam, and cefiderocol target resistant organisms [7]. Prevention relies on ventilator bundles; head elevation, daily sedation breaks, oral care with chlorhexidine, ulcer and DVT prophylaxis, and CDC measures [8].

**Fig. 2:**
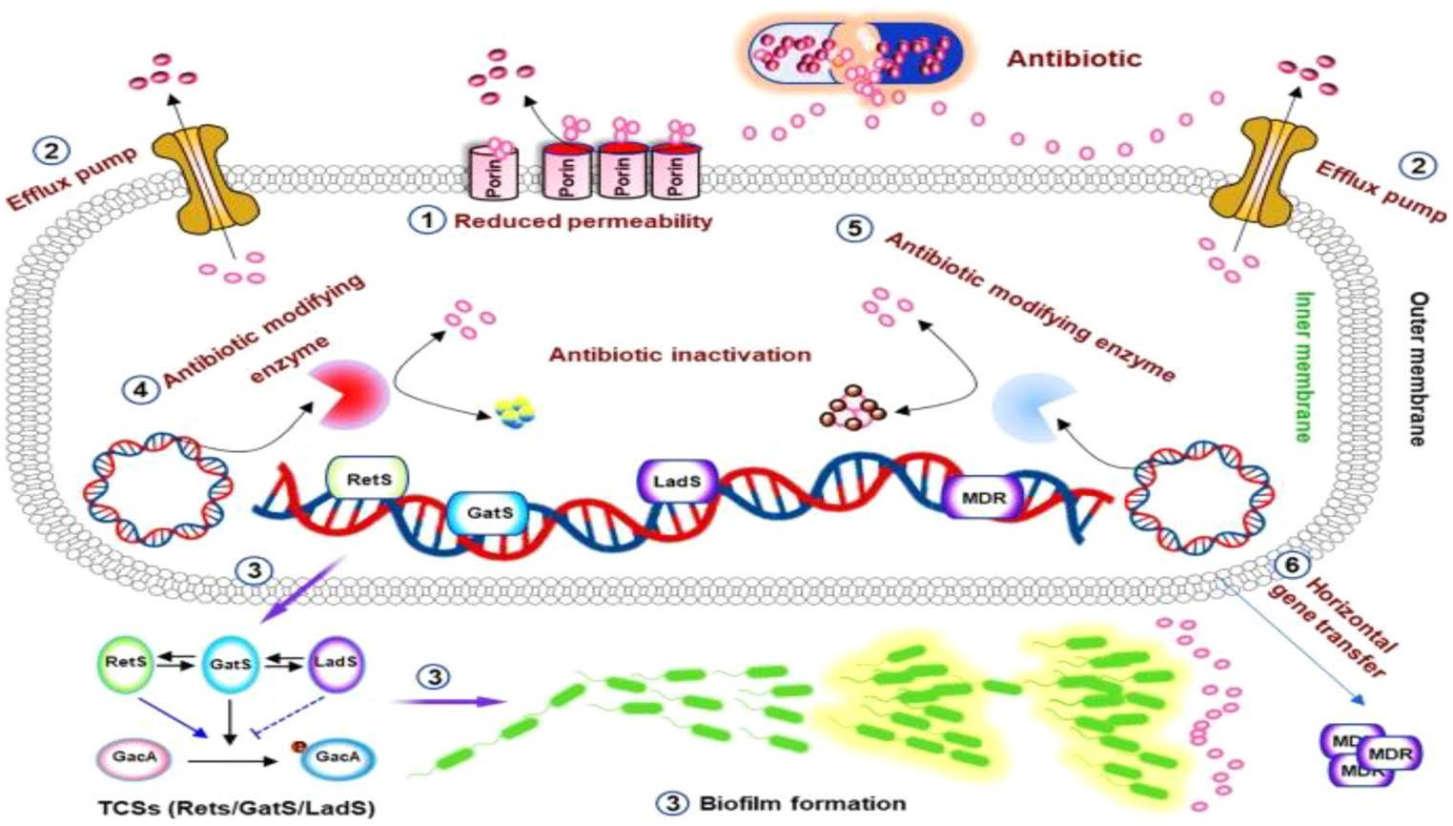
Mechanisms of antimicrobial resistance in *P. aeruginosa* [11]

Among the causative agents of VAP, *P. aeruginosa* is particularly significant. This opportunistic Gram-negative bacillus thrives in moist hospital environments and is known for its intrinsic resistance to many antibiotics. Furthermore, *P. aeruginosa* causes infection through multiple virulence mechanisms: adhesins (pili, flagella, LPS, alginate) for attachment and immune activation; potent secreted toxins and enzymes such as Exotoxin A, elastases, phospholipase C, pyocyanin; and the Type III secretion system. Quorum sensing and strong biofilm formation further enhance persistence and resistance [9, 10]. Clinically, it is a major cause of HAIs, including VAP, burn wound infections, surgical site infections, and lower respiratory tract infections in cystic fibrosis. It exhibits intrinsic (low permeability, efflux pumps, β-lactamases), acquired (gene transfer, mutations), and adaptive (biofilm-related) resistance, making treatment difficult [11] Fig. 2. Management relies on antipseudomonal β-lactams (piperacillin/tazobactam, cefepime, ceftazidime, carbapenems), sometimes combined with fluoroquinolones or aminoglycosides, with newer agents (ceftolozane/tazobactam, ceftazidime/avibactam, cefiderocol) used for MDR strains [12].

According to CDC 2025, MDR *P. aeruginosa* is defined as non-susceptibility to at least one antibiotic in at least three classes of antipseudomonal antibiotics. The global rise in MDR *P. aeruginosa* strains has become a significant challenge in clinical settings, particularly in ICUs [13]. Subsequently, the World Health Organization (WHO) classified MDR *P. aeruginosa* as a critical-priority pathogen for research and development of new antimicrobials. Difficult-to-treat *Pseudomonas aeruginosa* (DTR-PA) implies resistance to not only carbapenems (such as meropenem, imipenem, and doripenem) but also piperacillin-tazobactam, ceftazidime, cefepime, ciprofloxacin, and levofloxacin [14]. This creates a need to use newer and costly antibiotic options (ceftolozane-tazobactam (CT), ceftazidime-avibactam (CZA), or to use older and/or potentially toxic options [14]. In this context, continuous surveillance of local antimicrobial susceptibility patterns is vital, particularly in high-risk areas such as ICUs. Understanding regional variations in resistance profiles can inform empirical therapy, optimize treatment outcomes, and guide antimicrobial stewardship efforts [15].

The emergence and rapid dissemination of MDR *P. aeruginosa* strains have significantly narrowed the spectrum of effective therapeutic options, leading to an increased reliance on older antibiotics, such as colistin, and the introduction of newer combination therapies, including ceftazidime/avibactam (CAZ/AVI) [16]. Colistin, a polymyxin antibiotic discovered in the 1940s, has re-emerged in recent years as a last-resort treatment for MDR Gram-negative infections. Despite its known nephrotoxic side effects, colistin remains an effective antimicrobial therapy, particularly when other options fail [17]. CAZ/AVI, a combination of a third-generation cephalosporin with a novel non-β-lactam/β-lactamase inhibitor. It has emerged as an effective and safe alternative for treating infections caused by MDR Gram-negative bacteria, extended-spectrum β-lactamase (ESBL), and some carbapenemase-producing organisms [18].

This study aims to compare the MIC of colistin by using the broth microdilution method and the MIC of ceftazidime-avibactam by using the E test against MDR *P. aeruginosa*, estimate the frequency of *P. aeruginosa* as a causative agent for VAP in ICU patients and their antibiotic susceptibility, and estimate the frequency of multi-drug resistance among the *P. aeruginosa* isolates.

## Subjects and methods

### Study design

This observational cross-sectional study was conducted over the period from January 2025 to June 2025 among 15 medical and surgical ICU patients with VAP at Kasr Alainy Hospital. Before the initiation of the study, ethical approval was obtained from the institutional review board (IRB) (code: MS-24-2025), and an informed consent was obtained from the patient’s guardian. A total of 120 endotracheal aspirates were collected from patients. This coincided with the timing of the mid-year update of the antibiotic stewardship policy, permitting six-monthly assessments following last-line antibiotic preservation.

### Materials

**Fig. 3:**
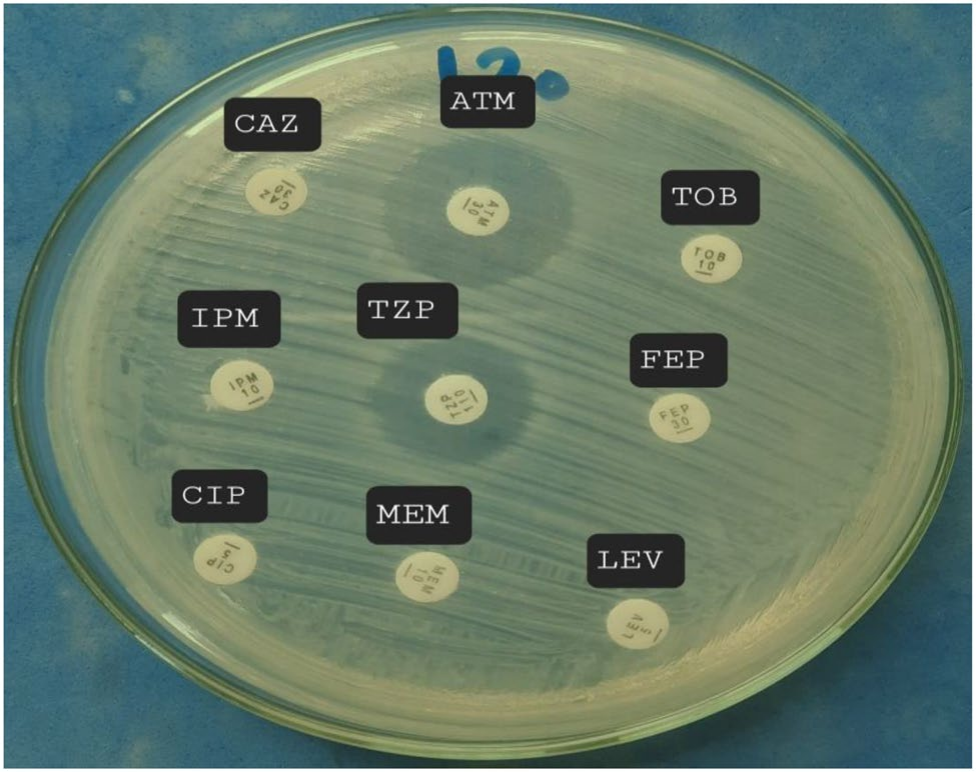
Disk diffusion method for *P. aeruginosa*

Patients’ demographic data, such as age, sex, comorbidities, and clinical data regarding the onset of VAP, were taken. The study was carried out in the Medical Microbiology and Immunology Department, Faculty of Medicine, Cairo University. Identification of isolates was confirmed by conventional methods, including colony morphology, Gram-stained smear, and biochemical reactions; oxidase tests, and triple sugar iron (TSI). Only isolates that were non-lactose fermenting on MacConkey’s agar no.3, Gram-negative bacilli, oxidase-positive with alkaline TSI, producing a greenish exopigment with a fruity odour, and capable of growth at 42 °C were identified as *P. aeruginosa* and selected for further testing [19]. All *P. aeruginosa* isolates were tested for antibiotic susceptibility by the Kirby-Bauer disc diffusion method Fig. 3 [19]. The following discs (Oxoid, UK) were applied **(CLSI**,** 2025)**: ciprofloxacin (CIP 5 µg), levofloxacin (LEV 5 µg), piperacillin/tazobactam (TZP 100/10µg), ceftazidime (CAZ 30 µg), cefepime (FEP 30 µg), tobramycin (TOB 10 µg), meropenem (MEM 10 µg), imipenem (IPM 10 µg), and aztreonam (ATM 30 µg). The diameter of each inhibition zone was measured in mm and was interpreted using CLSI, 2025 guidelines Table 1. Isolates that were not susceptible to at least one antibiotic in at least three classes of antipseudomonal antibiotics were considered as MDR strains **(CDC**,** 2025)**.

**Table 1:** Disk diffusion method breakpoints for *P. aeruginosa* (CLSI, 2025)

Antibiotic		Interpretation			
	Sensitive		Intermediate	Resistant	
CIP (5 µg)	≥ 25 mm		19–24	≤ 18 mm	
LEV (5 µg)	≥ 22 mm		15–21	≤ 14 mm	
TZP (100/10 µg)	≥ 22 mm		18–21	≤ 17 mm	
CAZ (30 µg)	≥ 18 mm		15–17	≤ 14 mm	
FEP (30 µg)	≥ 18 mm		15–17	≤ 14 mm	
TOB (10 µg)	≥ 19 mm		13–18	≤ 12 mm	
MEM (10 µg)	≥ 19 mm		16–18	≤ 15 mm	
IMP (10 µg)	≥ 19 mm		16–18	≤ 15 mm	
AZT (30 µg)	≥ 22 mm		16–21	≤ 15 mm	

Ciprofloxacin (CIP), levofloxacin (LEV), cefepime (FEP), ceftazidime (CAZ), imipenem (IPM), meropenem (MEM), tobramycin (TOB), piperacillin/tazobactam (TZP), aztreonam (AZT)

Determination of colistin MIC using broth microdilution method: Bacterial inoculum was prepared by adjusting the turbidity of the suspension to match the 0.5 McFarland standard, then diluted to 1:100 to obtain a final concentration of 1 × 10^6^ CFU/mL. A colistin stock solution of 2000 µg/mL was prepared and subsequently used, as outlined in CLSI 2025, to prepare serial dilutions over the range of 128 µg/mL to 0.25 µg/mL. A volume of 100 µl of each tested isolate was added to wells 1 through 10 in each row that contained serial colistin dilutions starting from 64 µg/mL to 0.125 µg/mL with a final bacterial concentration of 5 × 10^5^ CFU/mL and a final volume of 200 µl in each well. MICs were determined by turbidity and confirmed by measuring the optical density at 600 nm using a spectrophotometer (Stat Fax 2100, USA). The results were interpreted according to CLSI breakpoints for *P. aeruginosa* **(CLSI**,** 2025)** Table 2.

**Table 2:** Interpretive criteria of colistin MICs **(CLSI**,** 2025)**

Antibiotic	Interpretation		
	Sensitive	Intermediate	Resistant
Colistin	-------------	≤ 2 µg/ml	≥ 4 µg/ml

**Fig. 4:**
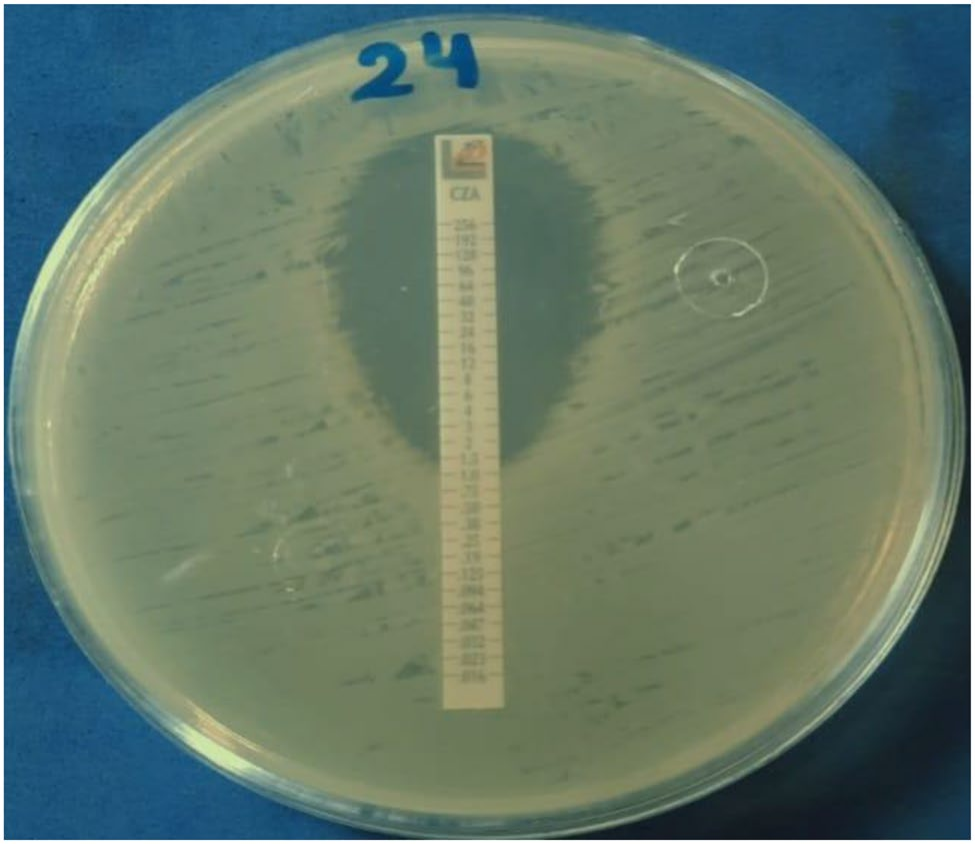
E test for determination CAZ/AVI MIC

Determination of ceftazidime avibactam MIC using E test for MDR *P. aeruginosa*: CAZ/AVI E test strips (Liofilchem, Italy) were placed on the inoculated plates and incubated aerobically at 37 °C for 18 h Fig. 4 [20]. The MIC was read at the intersection of the lower part of the ellipse-shaped growth inhibition area with the test strip and was interpreted using **CLSI**,** 2025** guidelines Table 3.

**Table 3:** Interpretive criteria of CAZ/AVI MIC **(CLSI**,** 2025)**

Antibiotic	Interpretation		
	Sensitive	Intermediate	Resistant
CAZ/AVI	≤ 8/4 µg/ml	----------	≥ 16/4 µg/ml

### Statistical analysis

Data was coded and entered using the statistical package for the Social Sciences (SPSS) version 28 (IBM Corp., Armonk, NY, USA). Data was summarized using mean, standard deviation, minimum and maximum in quantitative data and using frequency (count) and relative frequency (percentage) for categorical data. For comparison of paired measurements within each patient, the non-parametric Marginal Homogeneity test was used [21]. P-values less than 0.05 were considered statistically significant.

## Results

**Fig. 5:**
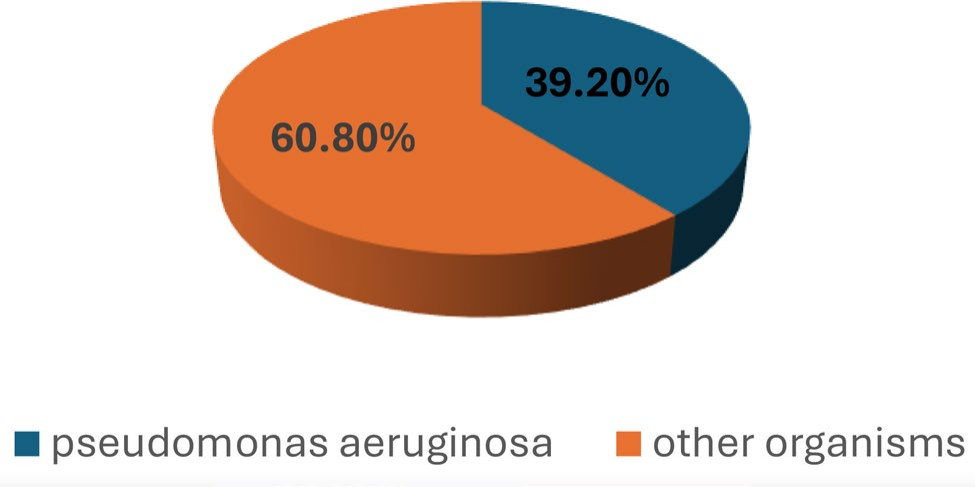
Distribution of *P. aeruginosa* isolates as a causative agent for VAP

In the present study, a total of 120 ICU patients with VAP were included. Among them, 47 (39.2%) patients had VAP episodes caused by *P. aeruginosa* Fig. 5.

As summarized in Table 4, the mean age of the patients was 67.7 years. Male patients were nearly double the females. Late-onset VAP occurred in most patients (85.1%), while early-onset VAP occurred in 14.9% patients. 38.3% of patients had hypertension, and 34% were diagnosed with diabetes mellitus.

**Table 4:** Characteristics of *P. aeruginosa* VAP patients

Parameters	No = 47 (100%)
Age, mean ± SD (years)	67.7 ± 9.98 years
Female sex	16 (33.0%)
Male sex	31 (66.0%)
Hypertension	18 (38.3%)
Diabetes	16 (34%)
Coronary heart disease	10 (21.3%)
Chronic kidney disease	5 (10.6%)
Chronic liver disease	7 (14.9%)
Early-onset VAP	7 (14.9%)
Late-onset VAP	40 (85.1%)

**Fig. 6:**
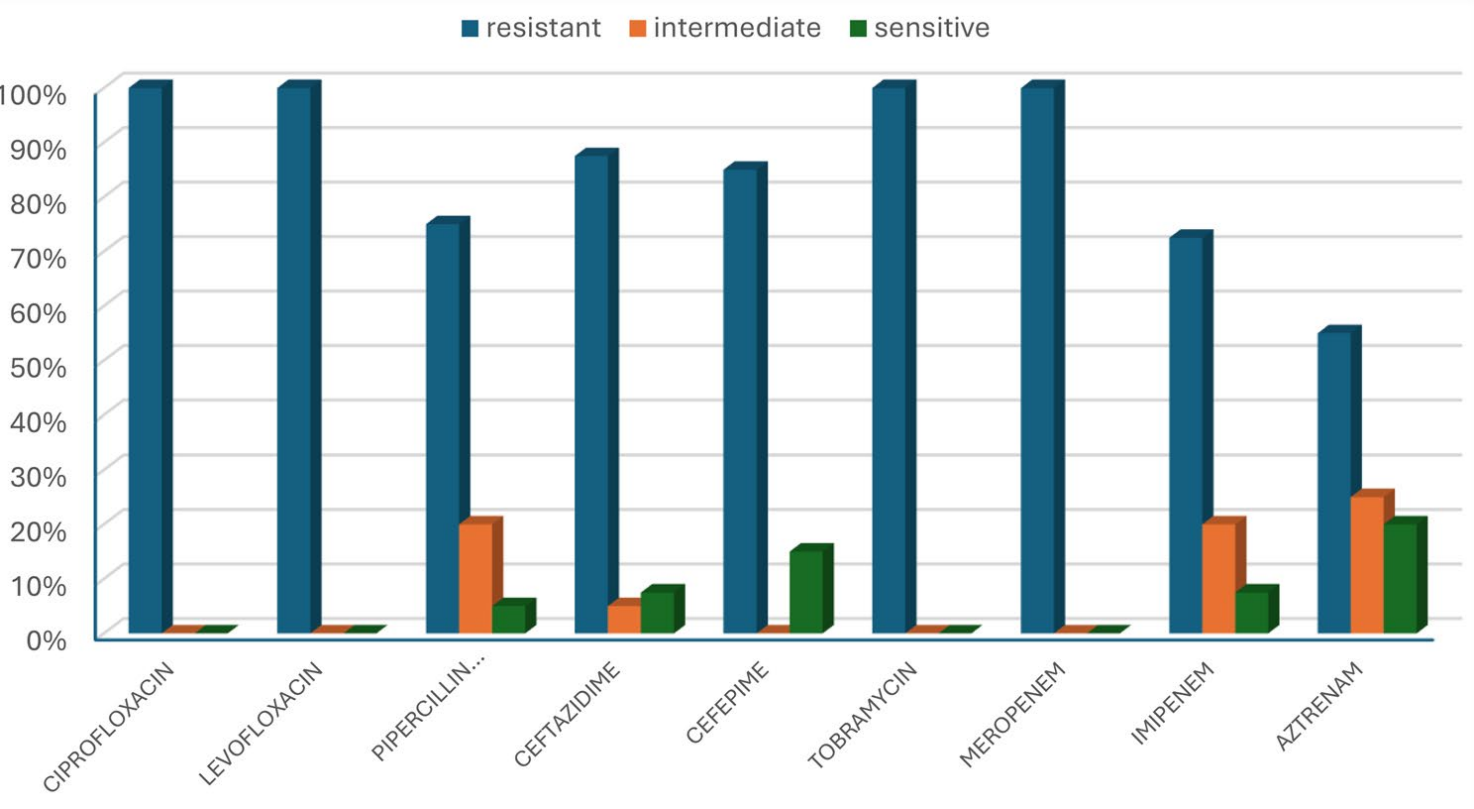
Antibiotic susceptibility profile of MDR *P. aeruginosa*

Of the total number of *P. aeruginosa* isolates, 85.1% were identified as MDR. 100% exhibited complete resistance to levofloxacin, ciprofloxacin, meropenem and tobramycin. 87.5% of isolates were resistant to ceftazidime, and 85.0% were resistant to cefepime. While lower resistance to piperacillin/tazobactam, imipenem and aztreonam were found to be 75.0%, 72.5% and 55%, respectively Fig. 6.

**Fig. 7:**
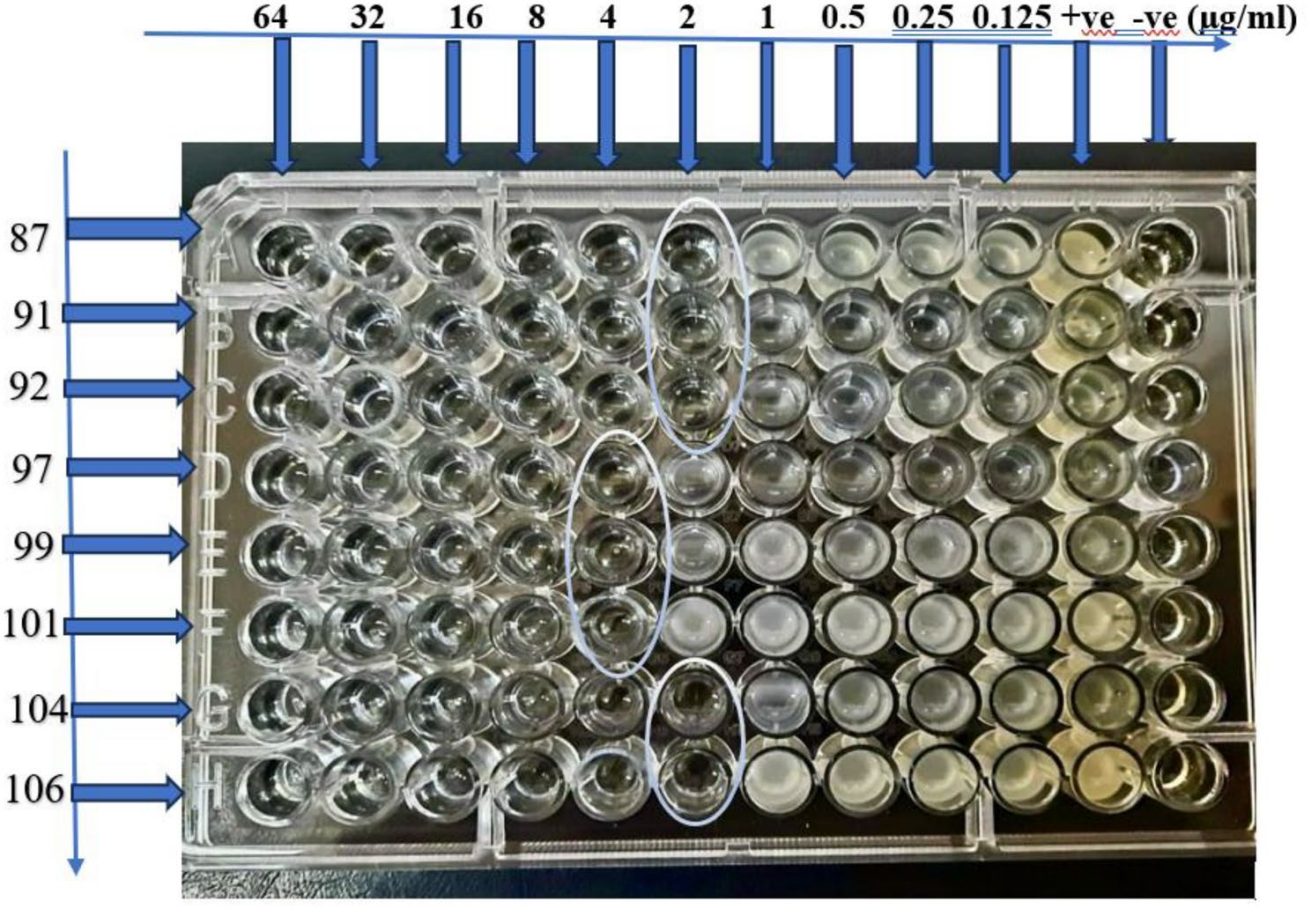
Detection of colistin MIC for MDR *P. aeruginosa* isolates using broth microdilution method showing MIC = 2 at sample 87, 91, 92, 104 and106 while MIC = 4 µg/ml at sample 97, 99 and 101

**Fig. 8:**
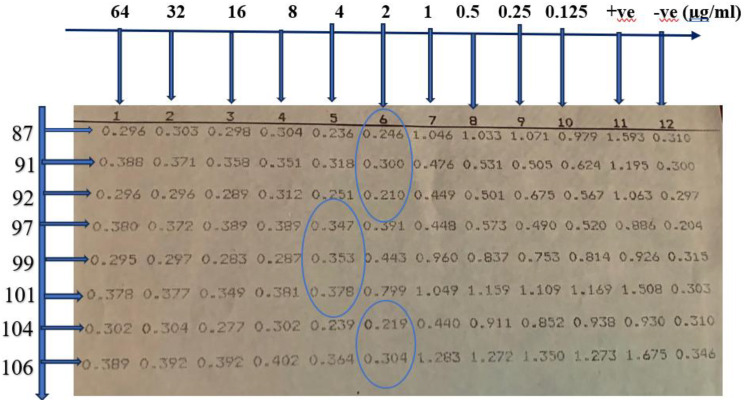
Detection of colistin MIC for MDR *P. aeruginosa* isolates by using spectrophotometer, showing MIC = 2 µg/ml at sample 87, 91, 92, 104 and106 while MIC = 4 µg/ml at sample 97, 99 and 101

**Fig. 9:**
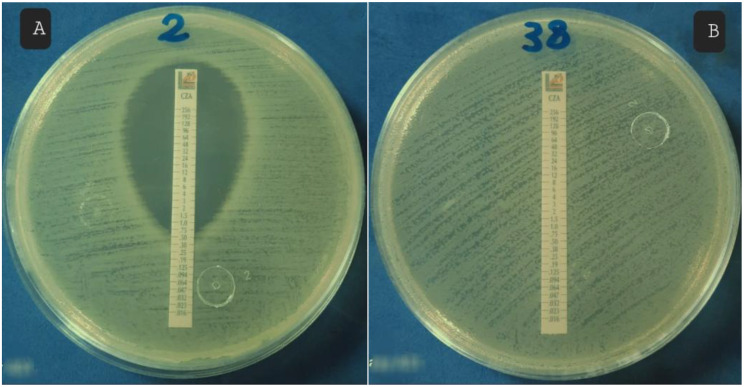
Detection of CAZ/AVI MIC for MDR *P. aeruginosa* isolates using E-test: **A**: susceptible with MIC = 0.75 µg/ml. **B**: resistant with MIC > 16 µg/ml

**Fig. 10:**
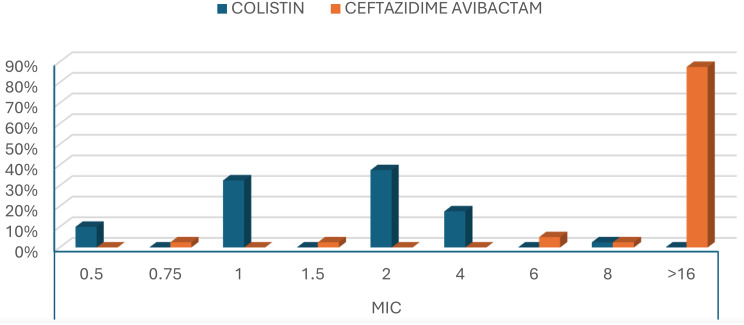
MIC values of colistin and CAZ/AVI for MDR *P.* *aeruginosa*

**Fig. 11:**
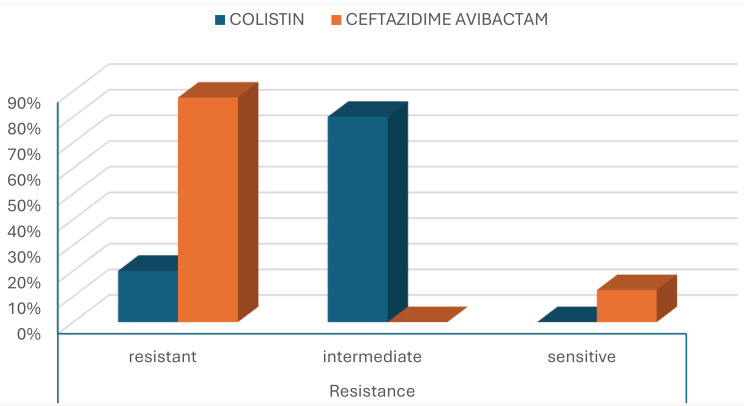
Comparing the antimicrobial activity of colistin and CAZ/AVI against MDR *P. aeruginosa*

Colistin showed a significantly higher antimicrobial activity (susceptibility, 80%; The MIC₅₀ and MIC_90_, 2 µg/mL and 4 µg/mL, respectively) against MDR *P. aeruginosa* isolates, Figs. 7 and 8, compared to CAZ/AVI (susceptibility, 12.5%; The MIC₅₀ and MIC_90_ >16 µg/ml), Figs. 9 and 10, with a statistically significant difference between them (*P-value* < 0.01), Table 5; Fig. 11.

**Table 5:** Comparing the antimicrobial activity of colistin and CAZ/AVI against MDR *P. aeruginosa*

N0. = 40		Colistin		CAZ/AVI		*P-value*
		**N0.**	**%**	**N0.**	**%**	
Results	Sensitive	---	----	5	12.5%	< 0.01
	Intermediate	32	80%	---	---	
	Resistant	8	20%	35	87.5%	

Ceftazidime avibactam (CAZ/AVI)

*P value* < 0.05 is considered significant

## Discussion

Our current study revealed that *P. aeruginosa* was responsible for 39.2% of VAP cases. Similarly, studies reported that 37% of VAP was caused by *P. aeruginosa* [22–24]. On the other hand, *P. aeruginosa* was a causative agent for VAP in only 6.4% and 8.9%, according to different studies [25, 26]. This figure may be attributed to that samples were taken from 15 medical and surgical ICUs after noticing a sharp increase in infection rates. This was part of updating antibiotic policies with a recommendation to continue the study to determine whether it has turned into an outbreak or endemic, and a recommendation to adhere to antibiotic policies according to the latest antibiogram. Most patients had late-onset VAP (85%), which is corroborated by the previous in vitro studies in 2015 and 2022 [27, 28]. The predominance of *P. aeruginosa* in late-onset VAP may be due to several factors as prolonged hospitalization and mechanical ventilation, colonization with hospital-acquired organisms, and prolonged antibiotic exposure [29]. In the current study, 85.1% of all *P. aeruginosa* isolates were identified as MDR, in agreement with the previous in vitro study in 2017 (prevalence rates 87.5% of MDR *P. aeruginosa*) [30]. In contrast, significantly lower MDR *P. aeruginosa* rates were observed in the USA and Europe (15.3% and 11.8%, respectively) [31, 32]. The wide variations in the epidemiology of MDR *P. aeruginosa* across countries are likely influenced by comparable antibiotic-prescribing practices [33]. The antibiotic susceptibility profile of our isolates revealed a notably high resistance rate to many tested antibiotics (100%, 87.5%, and 72.5% resistance rates to quinolones, cephalosporins, and carbapenems, respectively). Similarly, other studies in Egypt, Iran, and Saudi Arabia reported high resistance rates among *P. aeruginosa* isolates [34–36]. In contrast, much lower resistance rates were observed in studies in the USA and Switzerland [31, 37]. Acquisition of MDR *P. aeruginosa*. can be explained by prior exposure to broad-spectrum antibiotics (a key selection pressure), prior and prolonged ICU/hospital stay, prolonged mechanical ventilation, and use of invasive devices.

In the current study, our MDR *P. aeruginosa* isolates exhibited an 80% susceptibility rate to colistin, in agreement with previous in vitro studies in 2022 in Egypt and Vietnam [38, 39]. However, a lower susceptibility rate of (62.5%) was documented by the previous in vitro study in 2017, highlighting regional variability in colistin efficacy against MDR *P. aeruginosa* [30]. The MIC₅₀ and MIC₉₀ values for colistin in our study were 2 µg/mL and 4 µg/mL, respectively. In comparison, a previous in vitro study reported a susceptibility rate of 76%, with the same MIC₅₀ of 2 µg/ml but a markedly higher MIC₉₀ of 32 µg/ml, reflecting a broader distribution of resistance [40]. While a study in Qatar reported a susceptibility rate of 96.5%, with MIC₅₀ and MIC₉₀ values of 1.5 µg/mL and 2 µg/mL, respectively [41]. The elevated MIC₉₀ in our findings likely reflects a relatively lower susceptibility rate. It is generally observed that when susceptibility falls below 90%, the MIC₉₀ tends to increase, shifting the MIC distribution curve toward higher values. The high colistin susceptibility observed among MDR *P. aeruginosa* in our study may be due to colistin being reserved as a last-line option for severe, refractory cases, especially given its nephrotoxicity.

Recently, CAZ/AVI has emerged as an effective and safe alternative for treating infections caused by MDR *P. aeruginosa* [18]. Our isolates exhibited a low susceptibility rate to CAZ/AVI of (12.5%), supported by two previous studies in the USA and Egypt (26.3% and 23.5%, respectively) [42, 43]. In contrast, higher susceptibility rates have been reported in other studies in Germany and Poland (48.4% and 65.9% susceptibility rates to CAZ/AVI, respectively) [44, 45]. These findings reflect considerable geographic variability in CAZ/AVI efficacy, potentially influenced by local resistance patterns and antibiotic usage practices. The MIC₅₀ and MIC₉₀ values for CAZ/AVI were > 16 µg/mL, indicating limited in vitro activity against the tested MDR *P. aeruginosa* isolates, which is well supported by two other studies in 2023; The MIC₅₀ and MIC₉₀ values were > 16 µg/mL and were > 64 µg/mL, respectively [46, 47]. The reduced effectiveness of CAZ/AVI can be explained by its spectrum of activity, as it is effective against Ambler class A, class C, and some class D β-lactamases, but lacks activity against MBLs, which belong to class B [48].

In the current study, colistin demonstrated a significantly higher susceptibility rate (80%) against MDR *P. aeruginosa* isolates when compared to CAZ/AVI, which showed a notably low susceptibility rate of 12.5%. In support, high susceptibility rates for colistin (99.6%) and low susceptibility rates for CAZ/AVI (26.7%) were reported by different studies [42, 49, 50]. This can be attributed to the fact that colistin is reserved as a last-line option for severe refractory cases, especially since it is nephrotoxic. However, CAZ is frequently used in Kasralainy ICUs. CAZ/AVI needs Aztreonam to improve its penetration and outcome, which is not available in Kasralainy ICUs. Furthermore, CAZ/AVI resistance in *P. aeruginosa* is frequently multifactorial and may be caused by: carbapenemases not inhibited by avibactam as MBL, AmpC hyperproduction, efflux pump overexpression, and porin loss [51, 52]. In contrast, colistin targets the bacterial outer membrane (lipid A), so colistin resistance in *P. aeruginosa* most commonly arises via lipid A modification pathways and may also be presented as heteroresistance [53].

### Limitations

- The small sample size and short period, however, yielded meaningful analysis.
- Low susceptibility of CAZ/AVI needs molecular confirmation (e.g., MBL gene detection, *ampC* sequencing, efflux/porin assessment) [51, 52].
- Colistin has major clinical constraints even when “susceptible.”
- Systemic polymyxins are limited by dose-limiting nephrotoxicity, complex pharmacokinetics/pharmacodynamics, and heteroresistance. Dosing should be optimized based on renal function [54].

### Future directions

- Broaden surveillance: over longer periods and ideally multiple centers to confirm generalizability.
- Expand local susceptibility testing to other agents recommended for DTR-PA: test additional modern anti-pseudomonal/anti-MDR options (as locally available) to build a clinically useful pathway, such as ceftolozane–tazobactam, imipenem-cilastatin-relebactam, cefiderocol.
- Clinical correlation: Link in-vitro susceptibility patterns with patient severity and outcomes to support clinical decision-making.
- Several studies have suggested that liposome-based co-delivery is a promising strategy to combat colistin-resistant *P. aeruginosa* and improve treatment outcomes. This approach enhances targeted delivery and reduces systemic exposure [55].
- Add molecular characterization: Use resistance gene detection (e.g., multiplex platforms) to molecularly profile CAZ/AVI-resistant isolates, focusing on carbapenemases (especially MBL) and relevant chromosomal mechanisms (e.g., AmpC dysregulation, efflux upregulation, porin changes).
- Consider carbapenem-sparing approaches in future studies, as a potential strategy to reduce the MDR selection pressure.
- A combination therapy of aztreonam with CAZ/AVI has been proposed in recent literature, especially in settings with high MBL prevalence, where CAZ/AVI activity may be compromised [56].

## Conclusions

From this study, we conclude that *P. aeruginosa* was a common cause of VAP among ICU patients (39.2%), with most cases being late onset (85.1%). 85% of *P. aeruginosa* isolates were MDR, showing 100% resistance to key antipseudomonal agents, including fluoroquinolones, carbapenems, and aminoglycosides. Colistin demonstrated significantly higher in vitro activity (80%) against MDR *P. aeruginosa* isolates with MIC₅₀ and MIC₉₀ values of 2 µg/ml and 4 µg/ml, respectively. CAZ/AVI demonstrated much lower activity (12.5%) with both MIC₅₀ and MIC₉₀ exceeding 16 µg/ml. The difference in susceptibility between colistin and CAZ/AVI was statistically significant (*p* < 0.01). Colistin is considered a last-line option in the treatment of MDR *P. aeruginosa* VAP, though its potential toxicity must be carefully considered.

## Supplementary Information

Below is the link to the electronic supplementary material.

Supplementary Material 1

## Data Availability

[The data that support the conclusions of this article are included in this published article. The bacteria data, including the resistance, VAP onset, and any other comorbidities, can be accessed.](MDR%20Pseudomonas,%20data%20sheet.xlsx).

## Abbreviations

ATM: Aztreonam
BAL: Bronchoalveolar lavage
CAZ: Ceftazidime
CAZ/AVI: Ceftazidime-avibactam
CDC: Centers for disease control and prevention
CFU: Colony-forming unit
CIP: Ciprofloxacin
CLSI: The clinical laboratory standards institute
CT: Ceftolozane-tazobactam
DVT: Deep venous thrombosis
DTR-PA: Difficult-to-treat *Pseudomonas aeruginosa*
ESBL: Extended-spectrum β-lactamase
FEP: Cefepime
HAI: Hospital acquired infections
ICU: Intensive care unit
IPM: Imipenem
IRB: The institutional review board
LEV: Levofloxacin
MBL: Metallo-β-lactamases
MDR: Multi-drug resistance
MEM: Meropenem
MIC: Minimal inhibitory concentration
MRSA: Methicillin-resistant staphylococcus aureus
MSSA: Methicillin-sensitive Staphylococcus aureus
SPSS: The statistical package for the social sciences
TOB: Tobramycin
TSI: Triple sugar iron
TZP: Piperacillin-tazobactam
WHO: The World Health Organization
VAP: Ventilator-associated pneumonia
