# Supplementary figures and images for: Comparison between the antimicrobial activity of colistin and ceftazidime-avibactam against multidrug-resistant Pseudomonas aeruginosa isolated from patients with ventilator-associated pneumonia in intensive care units of Kasr Al-Ainy hospitals
Source: BMC Infect Dis. 2026 Mar 24;26:741. doi: 10.1186/s12879-026-12991-7 (PMC13064402; doi:10.1186/s12879-026-12991-7)

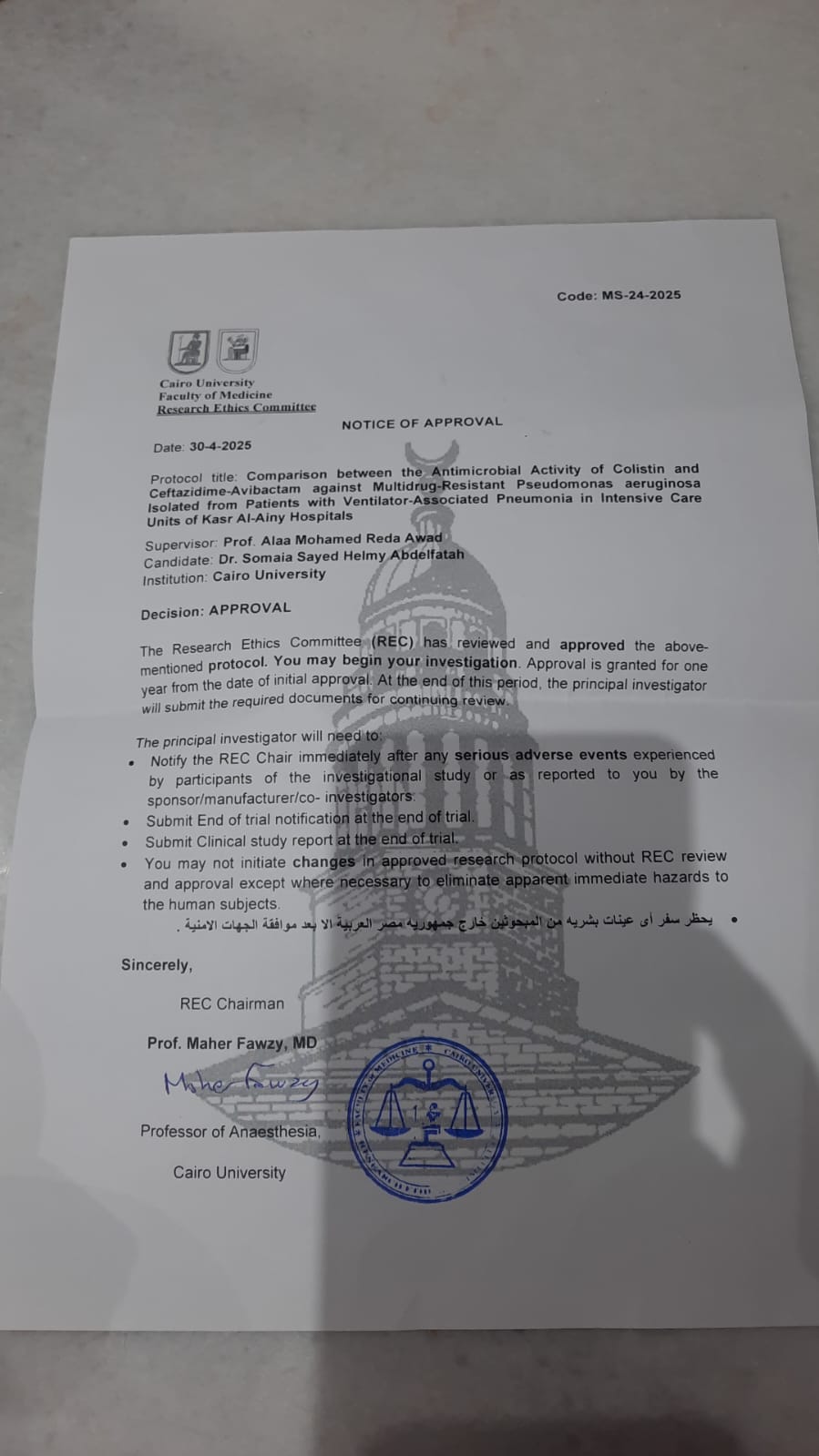

Supplement: Supplementary file 1 — Supplementary Material 1 [file 12879_2026_12991_MOESM1_ESM.jpeg]
